# Aperiodic Activity Indexes Neural Hyperexcitability in Generalized Epilepsy

**DOI:** 10.1523/ENEURO.0242-24.2024

**Published:** 2024-09-03

**Authors:** Markus Kopf, Jan Martini, Christina Stier, Silke Ethofer, Christoph Braun, Yiwen Li Hegner, Niels K. Focke, Justus Marquetand, Randolph F. Helfrich

**Affiliations:** ^1^Hertie Institute for Clinical Brain Research, University Medical Center Tübingen, Tübingen 72076, Germany; ^2^Graduate Training Centre of Neuroscience, International Max Planck Research School, University of Tübingen, Tübingen 72076, Germany; ^3^Institute for Biomagnetism and Biosignal Analysis, University of Münster, Münster 48149, Germany; ^4^Department of Neurosurgery, University Medical Center Tübingen, Tübingen 72076, Germany; ^5^Magnetoencephalography (MEG) Center, University of Tübingen, Tübingen 72076, Germany; ^6^CIMeC Center for Mind/Brain Sciences, University of Trento, Rovereto 38068, Italy; ^7^Department of Neural Dynamics and Magnetoencephalography, Hertie Institute for Clinical Brain Research, University of Tübingen, Tübingen 72076, Germany; ^8^Department of Neurology, University Medical Center Göttingen, Göttingen 37075, Germany; ^9^Department of Neurology and Epileptology, University Medical Center Tübingen, Tübingen 72076, Germany; ^10^Institute for Modelling and Simulation of Biomechanical Systems, University of Stuttgart, Stuttgart 70569, Germany

**Keywords:** 1/f spectral slope, alpha oscillations, aperiodic activity, interictal discharges, large-scale hyperexcitability, nonoscillatory activity

## Abstract

Generalized epilepsy (GE) encompasses a heterogeneous group of hyperexcitability disorders that clinically manifest as seizures. At the whole-brain level, distinct seizure patterns as well as interictal epileptic discharges (IEDs) reflect key signatures of hyperexcitability in magneto- and electroencephalographic (M/EEG) recordings. Moreover, it had been suggested that aperiodic activity, specifically the slope of the 1/*ƒ^x^* decay function of the power spectrum, might index neural excitability. However, it remained unclear if hyperexcitability as encountered at the cellular level directly translates to putative large-scale excitability signatures, amenable to M/EEG. In order to test whether the power spectrum is altered in hyperexcitable states, we recorded resting-state MEG from male and female GE patients (*n* = 51; 29 females; 28.82 ± 12.18 years; mean ± SD) and age-matched healthy controls (*n* = 49; 22 females; 32.10 ± 12.09 years). We parametrized the power spectra using FOOOF (“fitting oscillations and one over *f*”) to separate oscillatory from aperiodic activity to directly test whether aperiodic activity is systematically altered in GE patients. We further identified IEDs to quantify the temporal dynamics of aperiodic activity around overt epileptic activity. The results demonstrate that aperiodic activity indexes hyperexcitability in GE at the whole-brain level, especially during epochs when no IEDs were present (*p* = 0.0130; *d* = 0.52). Upon IEDs, large-scale circuits transiently shifted to a less excitable network state (*p* = 0.001; *d* = 0.68). In sum, these results uncover that MEG background activity might index hyperexcitability based on the current brain state and does not rely on the presence of epileptic waveforms.

## Significance Statement

It had long been suspected that electric brain activity is systematically altered in hyperexcitability disorders, such as epilepsy. To date, it remained unclear how pathologic aperiodic activity can be quantified. Kopf et al. demonstrate that aperiodic MEG activity indexes neural hyperexcitability, especially when epileptic discharges were absent; hence, providing a novel noninvasive biomarker that possibly reflects neural excitability at the level of whole-brain recordings.

## Introduction

Hyperexcitability, a state that renders neurons more likely to fire an action potential is the defining neurophysiological feature of epilepsy, where neural circuits are prone to generating spontaneous and excessive electrical activity, which can lead to seizures (Rao and Lowenstein, 2015). Epilepsy can result from various factors, such as genetic mutations or structural abnormalities. In addition to manifest seizure activity, interictal epileptic discharges (IEDs) constitute the electrophysiological key signature of hyperexcitability in the human magneto- or electroencephalogram (M/EEG).

However, the temporal variability and intermitted nature of IEDs pose a diagnostic challenge in assessing interictal M/EEG recordings (Steinhoff et al., 2013). Clinical reports typically focus on the presence and distribution of neural oscillations that are clearly discernible in the time domain, such as delta (<4 Hz), alpha (8–12 Hz), or beta waves (13–35 Hz). In addition to prominent neural oscillations, the EEG is also characterized by the presence of nonoscillatory aperiodic activity (Smith, 2005), which is not consistently evaluated in clinical practice (Minthe et al., 2020). Previously, it has been argued that aperiodic activity in M/EEG may also exhibit systematic aberrations in epilepsy (Staba and Worrell, 2014). Electrophysiological aperiodic activity follows a complex 1/*ƒ^x^* scaling law, where the exponent *x* of the decay function (also termed the spectral slope when plotted in log–log space) typically ranges from 2 to 4 in empirical recordings (Freeman and Zhai, 2009; Miller et al., 2009; Buzsáki et al., 2012; He, 2014; Lendner et al., 2020). Recently, novel computational models indicated that the spectral slope in the range between 30 and 50 Hz might index the balance of excitation and inhibition (*E*/*I* balance) of neural circuits (Gao et al., 2017; Chini et al., 2021). Specifically, a relative shift toward excitation is associated with a flattening of the spectral slope, while a steepening predicts a shift toward inhibition. However, empirical evidence for this hypothesis remains sparse and is often indirect, e.g., a steepening of the spectral slope has been observed during sleep and under general anesthesia (Colombo et al., 2019; Lendner et al., 2020), while arousals and task engagement flatten the spectral slope (Waschke et al., 2021). To date, it remains unknown if aperiodic activity in clinically manifest hyperexcitability disorders, such as epilepsy, is systematically altered.

Here, we directly tested the model predictions in a heterogeneous patient cohort that suffered from either idiopathic generalized epilepsy (IGE), genetically generalized epilepsy (GGE), or genetic epilepsy with febrile (i.e. “feverish”) seizures (GEFS+). IGE, GGE, and GEFS+ constitute a phenotypically and genetically heterogeneous patient population, which is often of polygenic inheritance; albeit, monogenetic causes, such as specific pathologies in voltage-gated sodium channels or at the level of synaptic transmission, have been described (Catterall et al., 2010; Wolking et al., 2019).

The goal of the study was threefold. First, we tested if hyperexcitability as assessed by IEDs can be inferred from the nonoscillatory aperiodic activity of whole-head neural recordings. We predicted a flattening of the spectral slope in the patient cohort as compared with age-matched controls. Second, we determined whether aperiodic activity provides unique or redundant information to oscillatory brain activity. Specifically, parieto-occipital alpha oscillations in healthy participants have been interpreted to reflect functional inhibition during visuospatial attention and working memory tasks (Klimesch et al., 2007; Jensen and Mazaheri, 2010); hence, increased alpha activity indexes less excitable brain states and might thereby regulate selective information processing (Vaudano et al., 2017). Thus, we hypothesized that, if nonoscillatory aperiodic activity yields unique information about excitability dynamics that cannot be inferred from oscillatory activity, it might constitute a promising clinically relevant MEG biomarker to infer large-scale excitability. Lastly, we tested if IEDs also modulate neural excitability as indexed by the spectral slope. Hence, we assessed if the spectral slope systematically changes from before to after an IED. To address these questions, we recorded up to 30 min eyes-closed resting–state recordings from 275-channel whole–head MEG in a large cohort of GE patients and healthy controls.

## Materials and Methods

### Participants

We recruited a heterogeneous patient cohort that exhibited generalized epileptic discharges in the MEG. In total, 57 patients (30.35 ± 12.90 years; mean ± SD; range, 7–64 years; 33 females) as well as 60 age-matched (*p* = 0.2772; *d *= 0.20; *t*_115_ = 1.09; unpaired *t* test) healthy controls (32.90 ± 12.35 years; mean ± SD; range, 17–63 years; 29 females) took part in this study. All patients were recruited from the Department of Neurology and Epileptology at the University Medical Center Tübingen in Germany. Eleven controls (seven females, four males) and six patients (four females, two males) had to be excluded due to strong head movements, insufficient data (<60 trials; see below), or other technical issues. Hence, the final sample included 51 IGE/GGE/GEFS + patients (Extended Data Table 1-1; 28.82 ± 12.18 years; mean ± SD) and 49 healthy controls (32.10 ± 12.09 years; mean ± SD; *p* = 0.1799; *d* = 0.27; *t*_98 _ = 1.35). The final patient sample included five separate subgroups [juvenile absence epilepsy (JAE): *n* = 16; juvenile myoclonic epilepsy (JME), *n = *7; idiopathic/nonclassified: *n* = 9; *STX1B* mutations, *n* = 15; *SCN1A* mutations, *n* = 4]. All patients had a history of convulsions or seizures and exhibited generalized epileptiform discharges in the MEG. Most patients received antiseizure medications (ASMs), including valproate, levetiracetam, lamotrigine, or a combination of different medications (Extended Data Table 1-1). Note that some patients were seizure-free for >10 years without medication; hence, they did not fulfill the current ILAE criteria (International League Against Epilepsy Consortium on Complex Epilepsies, 2018) but continued to exhibit IEDs in MEG, potentially reflecting hyperexcitability (Stefanou et al., 2017), and were, therefore, included in the present study. The study and analyses were approved by the University Medical Center Tübingen (protocol numbers, 492/2018BO2 and 454/2022BO1) and conducted in accordance with the sixth Declaration of Helsinki. All patients provided written informed consent to participate in the study. For patients younger than 18 years old, their parents provided written informed consent for their children to participate in the study in accordance with the IRB approval.

### Experimental design and procedures

We obtained up to 30 min of eyes-closed, MEG resting–state recordings in supine position for every participant. Participants were instructed to close their eyes and move as little as possible while not falling asleep. We did not observe any seizures during the recordings. For source reconstruction, we obtained structural magnetic resonance imaging for all participants, except for three patients.

### MEG data acquisition

MEG recordings were performed at the MEG center in Tübingen, Germany, using a 275-channel whole–head CTF MEG system (VSM MedTech), which was placed in a magnetically shielded room (Vacuumschmelze). We obtained 30 min of resting-state recordings in all patients and up to 30 min for healthy controls (39 × 15 min, 15 × 30 min, 5 × 8 min, 1× unknown due to technical problems, which was later excluded). Note that only participants with at least 15 min of recordings were included for further analyses. We confirmed that the difference in recording length did not impact any of our analyses. Data were recorded using a minimal sampling rate of 585.9 Hz. In some instances, the sampling rate during the initial recordings was increased, but all recordings were subsequently downsampled by an integer number.

### Image data acquisition

Structural imaging was obtained at 3 Tesla on either a Magnetom Trio (A Tim System, Siemens Medical Solutions) or a Magnetom Prisma MRI scanner at the University Hospital Tübingen. We obtained high-resolution structural T1–weighted MRIs (MPRAGE; TE, 3.03 ms; TR, 2,300 ms; TI, 900; flip angle, 8°) using a 64-channel head coil with 1 mm^3^ isotropic resolution. Only images without significant artifacts were considered for subsequent processing. Individual MRIs were available for all participants except for three patients. For these individuals, we employed a template MRI as included in the FieldTrip toolbox (Oostenveld et al., 2011). In most of the cases, structural imaging (∼30 min duration) was performed on the same day as the MEG. Only in a few instances, structural imaging has been performed prior to or after the MEG recordings. The individual structural MRIs were then further segmented using FreeSurfer 6.0.0 (https://surfer.nmr.mgh.harvard.edu/) to reconstruct the individual subcortical and cortical regions (Fischl, 2012). To achieve anatomical alignment across individuals, we employed the surface mapper SUMA to reconstruct each surface (Saad and Reynolds, 2012). Surfaces were generated using a standard template as included in FreeSurfer.

### MEG data preprocessing

All analyses were conducted in MATLAB version R2020b (MathWorks) using the FieldTrip toolbox (Oostenveld et al., 2011) as well as the custom code. The time series data were demeaned, linearly detrended, and downsampled. In addition, a low-pass filter at 90 Hz and a high-pass filter at 0.1 Hz were applied. In four control participants, we observed very strong slow fluctuations, which were attenuated by means of a 1 Hz high-pass filter. To remove line and additional ambient noise, one notch filter was applied at 50 Hz and another one at 51.2 Hz. After filtering, the data were segmented into 10-s-long, nonoverlapping trials. Trials that contained substantial artifacts as well as noisy channels were removed after visual inspection. To provide a sufficient amount of IEDs and IED-free data, patients with <60 trials after visual artifact rejection were excluded from subsequent analyses. Finally, independent component analysis as implemented in the FieldTrip toolbox (method, *fastICA*; Hyvarinen, 1999) was employed to identify and remove heartbeat, eye movement or muscle artifacts.

### IED detection

IEDs were detected semiautomatically in sensor-level data using previously established algorithms (Gelinas et al., 2016; Helfrich et al., 2019). All cutoffs were chosen in accordance with recently published reports and were subsequently visually inspected by a neurologist. In brief, individual channels were filtered between 25 and 80 Hz, and the analytical amplitude was extracted from the Hilbert transform. The resulting traces were then *z*-normalized. Events were categorized as IEDs when the signal exceeded the mean by 3 standard deviations (SD) for a duration of >20 ms but <100 ms. The epoch was then time-locked to the IED peak. Subsequently, the segments that contained the IEDs (±2 s) were separated from the IED-free data (IED−). Nonoverlapping segments containing only a single IED ± 5 s were then utilized for the analyses reported in Figure 4. IED-free epochs were segmented into 10 s segments. One additional patient was removed at this analysis stage given an insufficient amount of IED-free data. This participant was excluded from the group comparisons (Figs. 2, 3) but remained in the IED analyses reported in Figure 4.

### Spectral analysis

Time series were transformed into the spectral domain by means of a fast Fourier transformation. We employed a multitaper approach (Mitra and Pesaran, 1999) based on discrete prolate Slepian sequences, using 39 tapers resulting in a frequency smoothing of ±2 Hz. Spectral estimates were computed from 1 to 45 Hz in 0.5 Hz steps and subsequently averaged across trials resulting in one power spectrum per channel for every participant.

### Estimation of aperiodic activity

In order to extract the aperiodic component from the signal, we applied the “fitting oscillations and one over *f*” (FOOOF) algorithm (Donoghue et al., 2020) for frequencies between 1 and 45 Hz. We parametrized the power spectra using the FOOOF algorithm with standard settings (peak width limits, [0.5 12]; no predefined peak number; min. peak height, 0; peak threshold, 2). All power spectra were initially parametrized using the “knee” mode, which fits a Lorentzian function with a parameter *k* that indicates the deflection point. However, in many instances, we did not observe a clear knee [controls, 33 ± 4.7 channels (mean ± SEM; range, 0–151 channel); IED+, 33.5 ± 8.2 channels (range, 0–263 channels); IED−, 37.4 ± 8.0 channels (0–224 channels)]. This case corresponds to a *k* = 0 and resulted in a linear fit, which is analogous to the “fixed” FOOOF mode. Hence, we refrained from further analyzing the knee parameter. Note that we display grand averages in all figures, which visually give the impression of the presence of a knee at the group level as the result of averaging. The variable presence of a clear knee in individual subjects and channels impeded subsequent analyses at the group level.

This analysis yielded the negative spectral slope parameter *χ* (the negative exponent of the 1/*f^x^* function), the *y*-intercept *c* and a time constant *k* representing the knee parameter. The aperiodic spectrum was defined as follows:

$$\mathrm{aperiodic}\;\mathrm{fit}=10^{c}\;*\;\frac{1}{\left(k+f^{\chi }\right)}.$$

Alpha power was defined as the average in the range from 8 to 12 Hz as calculated on the residuals.

### IED-locked spectral analysis

To obtain a time–frequency representation centered on the IEDs (±5 s), we applied a moving Hanning window (window size, 500 ms; step size, 50 ms) to obtain spectral estimates from 1 to 45 Hz in 1 Hz steps. We again separated oscillatory from aperiodic components using the FOOOF algorithm.

### Source localization

The preprocessed and cleaned data were projected into an MNI-aligned source space using linearly constrained minimum variance beamforming (Van Veen et al., 1997). For each participant, a single-shell leadfield was created using either the individual MRI (if available) or a standard MRI template (Nolte, 2003). The forward model was created using a common dipole grid (10 mm^3^ grid) in MNI space, warped onto a standard MRI template. The covariance matrix was then computed for every dataset separately. The spatial filter B was calculated based on the covariance matrix C (dimensions, channels × channels) resulting in the leadfield L (dimensions, channels × 3) as follows:

$$B=(L^{T}C^{-1}L{)}^{-1}L^{T}C^{-1}.$$

We analyzed activity at all grid points that were defined in the anatomical automatic labeling atlas as included in FieldTrip. After projection of the sensor-level data through the spatial filter, we received source localized data in the time domain for 1,459 grid points as defined by the atlas. These data were subsequently processed analogous to the sensor-level data.

### Statistical analysis

For statistical comparisons, we employed cluster-based permutation tests (Maris and Oostenveld, 2007) to correct for multiple comparisons as implemented in FieldTrip (Monte Carlo method; 1,000 iterations; maxsum criterion). Clusters were formed in space by thresholding two-tailed independent (Fig. 2*B*,*D*) or dependent (Figs. 3*B*,*D*, 4*B*,*D*) *t* tests at a critical alpha of 0.05. A permutation distribution was then created by randomly shuffling group or condition labels. The permutation *p* value was obtained by comparing the cluster statistic to the random permutation distribution. The clusters were considered significant at a critical two-tailed alpha of 0.05.

Correlations between alpha power and the spectral slope were computed on the average across all the channels within the significant clusters (Spearman's rank correlation considered significant at *p* < 0.05). Given that the correlation was always computed for two groups (HC and IED−; IED− and IED+), we employed a Bonferroni’s correction of the *p* value, which was multiplied by the number of tests (*N *= 2), to account for multiple comparisons. Note that the correlation *p* value reported throughout the manuscript refers to the corrected *p* value after Bonferroni’s correction.

To assess the effect of the five different patient subgroups (JAE; JME; idiopathic/nonclassified; *STX1B*; *SCN1A*) or the medication status (valproate, levetiracetam, lamotrigine, multiple medications, no medication, not available), we employed a nonparametric permutation approach to account for the low number of participants after these stratification procedures. In brief, we repeated a one-way analysis of variance (ANOVA) across the average of all channels within the significant cluster, as determined by cluster-based permutation testing between patients and controls (Fig. 2) or between pre- versus post-IED (Fig. 4), 1,000× after randomly shuffling the condition label, which yielded a distribution of surrogate *F* values. The observed *F* value was then compared with the distribution to obtain the respective *p* value.

Effect sizes were quantified by means of Cohen’s *d*, the correlation coefficient rho, or eta-squared (*η*^2^) in case of ANOVAs.

## Results

**Figure 1.**
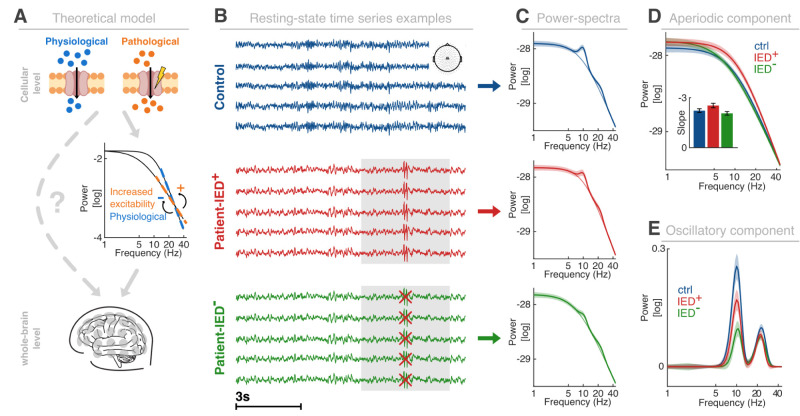
Experimental setup, theoretical model, and analytical approach. ***A***, Computational modeling to link cellular properties and/or pathologies to large-scale neural activity. Top, Two ion channels are illustrated under physiologic (left) and pathologic (right) conditions. Center, A computational model that links cellular properties to network level activity. A flattening of the spectral slope reflects increased excitability. Bottom, Illustration of whole-head MEG recordings at the sensor level. ***B***, Exemplary resting-state recordings from five central sensors (inset; single subject example) in the time domain. Top, Healthy control (ctrl). Center, Recording from a patient including the IEDs (IED+; gray box; Extended Data Table 1-1). Bottom, The same patient data after removing time segments containing IEDs (IED−). Note that only time segments around individual IEDs (±2 s) were removed for IED−, otherwise the data for IED+ and IED− are identical. ***C***, Grand average power spectra per group. The solid light line indicates the aperiodic 1/*f* decay function (see also panel ***D***). The same conventions as in panel ***B***. ***D***, Comparison of the aperiodic activity [quantified by the spectral slope; the inset illustrates the slope (mean ± SEM); for statistical quantification, see Figs. 2, 3] between groups. ***E***, Group-level comparison of the oscillatory residuals (after subtraction of the aperiodic component from the power spectrum shown in ***C***). Note the prominent peak in the canonical alpha band (∼10 Hz; quantification in Figs. 2, 3). Note that the *x*-axes in panel ***C–E*** are log-transformed. Lines and shaded areas indicate the mean and SEM.

We recorded whole-head, eyes-closed resting–state magnetoencephalography from a heterogeneous patient population suffering from idiopathic or genetically generalized epilepsies (IGE/GGE/GEFS+; *n* = 51; 28.82 ± 12.18 years; mean ± SD; Extended Data Table 1-1) as well as healthy, age-matched controls (*n* = 49; 32.10 ± 12.09 years; mean ± SD; *p* = 0.1799; *d* = 0.27; *t*_98 _= 1.35; unpaired *t* test; Fig. 1*A*). In a subset of patients, a selective mutation in a sodium channel was deemed responsible for the hyperexcitability syndrome after genetic testing, while in others polygenetic or unknown causes gave rise to the epilepsy syndrome. We included five separate subgroups (see Materials and Methods) and tested if hyperexcitability in the context of generalized epileptic discharges modulated macroscale signatures that are commonly thought to index neural excitability (Fig. 1*A*), namely, the spectral slope (Gao et al., 2017) and alpha oscillations (Klimesch et al., 2007; Jensen and Mazaheri, 2010). IEDs constitute a key signature of clinically relevant hyperexcitability in MEG recordings, in addition to manifest seizure activity. The diagnostic challenge is that interictal activity is often highly comparable between patients and controls upon visual inspection when IEDs are absent (Fig. 1*B*). Hence, the central question was whether the system-level signatures of hyperexcitability could reliably distinguish patients and controls, even when IEDs are not present. Therefore, we spectrally decomposed the time domain signals (Fig. 1*C*) to estimate aperiodic (Fig. 1*D*) and oscillatory activity separately (Fig. 1*E*). We then extracted the spectral slope parameter from the aperiodic power spectrum as the negative exponent of the 1/*f^x^* decay function. Critically, we divided the data into two groups: one group contains the entire recording, including all IEDs (IED*+*), while the other group consisted of data where the IEDs (±2 s) were removed (IED−). The model successfully parametrized the power spectra as indicated by the overall goodness-of-fit (>>99%; controls, 99.02 ± 0.1%; IED−, 99.31 ± 0.1%; IED+, 99.01 ± 0.11%; mean ± SEM). We did not observe a significant difference between the three groups (*F* = 2.02; *p* = 0.14; *η*^2 ^= 0.03; one-way ANOVA).

10.1523/ENEURO.0242-24.2024.t1-1 Table1-1 **Patient characteristics** Overview of the included patient cohort, which specifies sex, age, diagnosis, medication status and time from the last seizure. Abbreviations: GGE: genetic generalized epilepsy; IGE: idiopathic generalized epilepsy; GTCS: generalized tonic clonic seizures; JAE: juvenile absence epilepsy; JME: juvenile myoclonic epilepsy; FC: febrile convulsions; SE: status epilepticus; LEV: Levetiracetame; LTG: Lamotrigine; VPA: Valproate; ESX: Ethosuximide; ESL: Eslicarbazepine; TPM: Topiramate; STP: Stiripentol; CLB: Clobazam. Download Table1-1, DOCX file.

### The spectral slope and alpha oscillations track neural hyperexcitability

**Figure 2.**
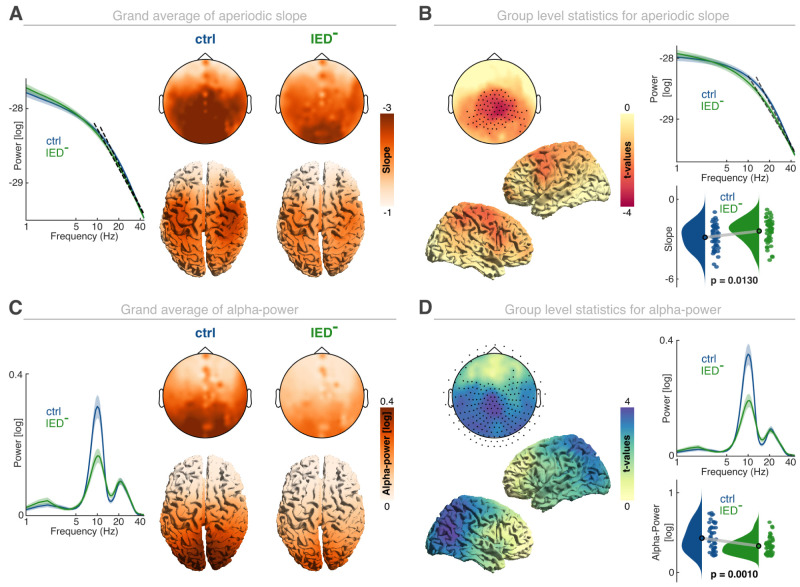
Modulation of large-scale excitability in GE patients. ***A***, Left, Grand average of the aperiodic component for controls and patients (IED−). Top row, Left, Sensor-level topography of the spectral slope in controls (left) and the IED− (right) group. Bottom row, Corresponding source space reconstruction of the spectral slope for controls (left) and patients (IED−, right). ***B***, Group-level statistics (ctrl vs IED−) contrasting the aperiodic slope between groups at sensor- (left-top panel; black dots highlight significant cluster sensors at cluster-corrected *p* < 0.05) and source-level (left-bottom panels). Top right, Mean aperiodic spectra across all significant channels. The dashed black line is the corresponding spectral slope. Bottom right, Distribution of spectral slopes in both groups. Individual dots reflect the average spectral slope within the significant cluster per participant. ***C***, Left, Grand average of the oscillatory residuals between both groups. Top row, Sensor-level alpha power for controls (left) and IED− patients (right). Bottom row, Source-level alpha power, analogous to the sensor-level topographies. ***D***, Left, Group-level statistics for the comparison of alpha power on the sensor- (top panel; black dots depict significant sensors at *p* < 0.05) and source-level (bottom panels). Right, Mean oscillatory residuals across all significant cluster channels (top) and the corresponding alpha power (bottom). Note that the *x*-axes of the power spectra are log-transformed. Lines and shaded areas indicate the mean and SEM.

First, we directly compared patients (IED*−*) with controls to test if systems-level signatures of hyperexcitability distinguish both groups, even when no salient epileptic activity in the form of epileptic discharges was present. For the spectral slope as a marker of aperiodic activity, we observed a comparable spatial distribution in patients and controls (Fig. 2*A*). The spectral slope was strongly flattened in patients in a large central cluster (Fig. 2*B*; *p* = 0.0130; *d *= 0.52; summed *t*_97 _= −242.79; cluster-based permutation test based on an unpaired *t* test; controls, −2.86 ± 0.13; IED−, −2.39 ± 0.13; mean ± SEM). This is in accordance with the idea that neural excitability is increased in GE, even when no IEDs are present. Across the patient sample, we observed a significant difference between the subgroups (*p* = 0.036; *η*^2 ^= 0.20; nonparametric permutation test) with generally flatter spectral slopes for the monogenetic patient population (*t*_(48) _= 2.23; *p* = 0.0303; *d* = 0.66; monogenetic, −2.01 ± 0.15; unpaired two-tailed *t* test; others, −2.48 ± 0.14; mean ± SEM). When compared with the control group, both groups exhibited significantly flatter spectral slopes (all *d* > 0.43; all *p* < 0.0324; unpaired one-tailed *t* test). We did not observe a significant effect of medication status on the spectral slope (*p* = 0.254; *η*^2 ^= 0.13; nonparametric permutation test). These findings indicate that, although neural excitability is elevated across the entire patient group as compared with healthy controls, the most substantial increase can be observed within monogenetic patient population.

Similarly, alpha oscillations showed the well known frontoparietal gradient with high alpha activity over posterior regions in both groups (Fig. 2*C*), which was strongly reduced in patients suffering from GE (Fig. 2*D*; *p* = 0.001; *d* = 0.73; summed *t*_(97) _= 742.88; controls, 0.29 ± 0.03; IED−, 0.16 ± 0.02; mean ± SEM), further supporting the idea that functional inhibition is attenuated in hyperexcitability disorders. We did not observe a significant difference between different patient groups (*p* = 0.136; *η*^2 ^= 0.14; nonparametric permutation test). Instead, alpha activity was comparable between the monogenetic patient cohort and the remaining patients (monogenetic, 0.16 ± 0.03; others, 0.16 ± 0.02; mean ± SEM). Moreover, we did not observe a significant effect of medication status on alpha activity (*p* = 0.73; *η*^2 ^= 0.06; nonparametric permutation test).

Notably, the negative correlation between the spectral slope and alpha power that was evident in healthy controls (rho = −0.53; *p* = 0.0002; Bonferroni-corrected Spearman rank correlation) was attenuated in GE patients (rho = −0.24; *p* = 0.1924). These results indicate that pathologies on the cellular or synaptic level differentially impact large-scale signatures of neural excitability and indicate that both metrics (spectral slope and alpha power) provide complementary information about states of hyperexcitability when IEDs were absent.

**Figure 3.**
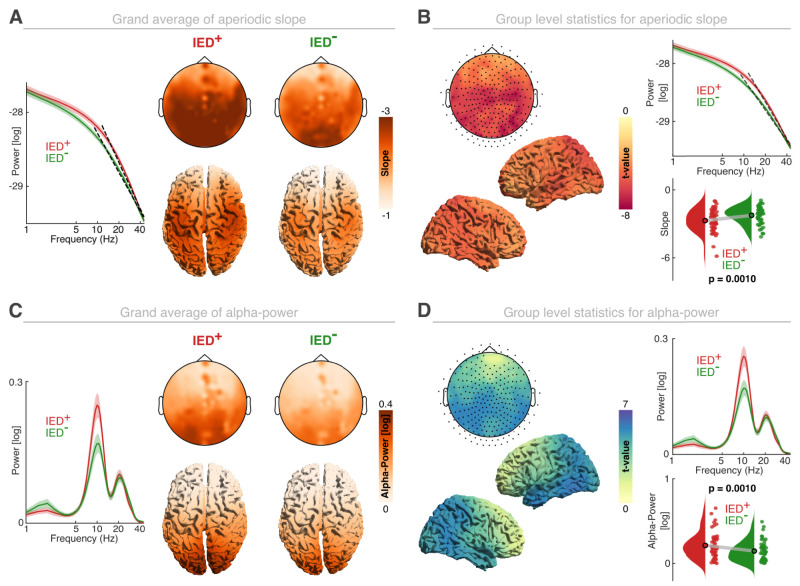
Interictal discharges modulate aperiodic and oscillatory activity. ***A***, Left, Grand average of the aperiodic component for IED+ and IED−. Top row, Sensor-level spectral slope for data with (left; IED+) and without (IED−) epileptic discharges. Bottom row, Corresponding source reconstruction. ***B***, Left, Group-level statistics for the comparison of the aperiodic slope at sensor- (top panel; black dots indicate significant channels in the cluster; *p* < 0.05) and source-level (bottom panels). Top right, Mean aperiodic activity across all significant cluster channels. The dashed black line is the corresponding spectral slope. Bottom right, Distribution of the spectral slope in both groups. Each dot represents the average value for one participant. ***C***, Left, Grand average of the oscillatory residuals for IED+ and IED− groups (mean ± SEM). Top row, Sensor-level alpha power for IED+ (left) and the IED− group (right). Bottom row, Corresponding source space reconstruction. ***D***, Left, Group-level statistics for the comparison of alpha power on sensor- (top panel; black dots indicate significant cluster channels at *p* < 0.05) and source-level (bottom panels). Right, Mean oscillatory residuals across significant cluster channels (top) and the corresponding alpha power (bottom). Note that the *x*-axes of the power spectra are log-transformed. Lines and shaded areas indicate the mean and SEM.

Next, we tested how the presence of salient epileptic activity in the form of IEDs impacts systems-level excitability markers. Therefore, we compared IED*+* and IED− epochs in GE patients. While the overall distribution of the spectral slope was comparable (Fig. 3*A*), it was evident that the spectral slope was significantly steeper when IEDs were present (Fig. 3*B*; *p* = 0.001; *d* = 1.08; summed *t*_(49) _= −1,557.20; cluster-based permutation test based on paired *t* tests; IED+, −2.68 ± 0.14; IED−, −2.21 ± 0.11; mean ± SEM). We did not observe systematic differences between IED+ and IED− epochs in the different patient subgroups (*p* = 0.21; *η*^2 ^= 0.12; nonparametric permutation test). For alpha oscillations, we again observed the expected frontoparietal gradient in both groups (Fig. 3*C*). Alpha power was significantly elevated across all areas in the data containing IEDs (IED+; Fig. 3*D*; *p* = 0.001; *d* = 0.80; summed *t*_(49) _= 1,267.67; IED+, 0.22 ± 0.02; IED−, 0.15 ± 0.02; mean ± SEM), but no significant difference was observed between the different patient subgroups (*p* = 0.19; *η*^2 ^= 0.12; nonparametric permutation test). Note that we also employed an additional bootstrapping procedure to control for the difference in trial numbers between IED− and IED+ (*p* < 0.001). To this end, we equated the trial numbers by random in 1,000 iterations. The bootstrapped estimates were highly correlated with the initially observed estimates (slope, rho = 0.99; *p* < 0.0001; alpha power, rho = 0.99; *p* < 0.0001). Hence, after bootstrapping, we observed highly comparable differences between IED+ and IED− groups.

In sum, this set of findings suggests that data that contain IEDs are characterized by a steepening of the spectral slope and increased alpha power, which jointly indicate increased inhibition. The spectral slope and alpha power modulation were not significantly correlated in either group (IED+, rho = −0.29; *p* = 0.0790; IED−, rho = −0.21; *p* = 0.2848; Bonferroni-corrected Spearman correlation), again indicating that both metrics provide nonredundant information about excitability dynamics. Under the assumption that IEDs index a hyperexcitable brain state, the opposite pattern (flatter slope and decreased alpha power) would have been expected. Thus, these findings raise the question if IEDs directly modulate both systems-level markers of neural excitability.

### Epileptic discharges transiently reduce neural hyperexcitability

**Figure 4.**
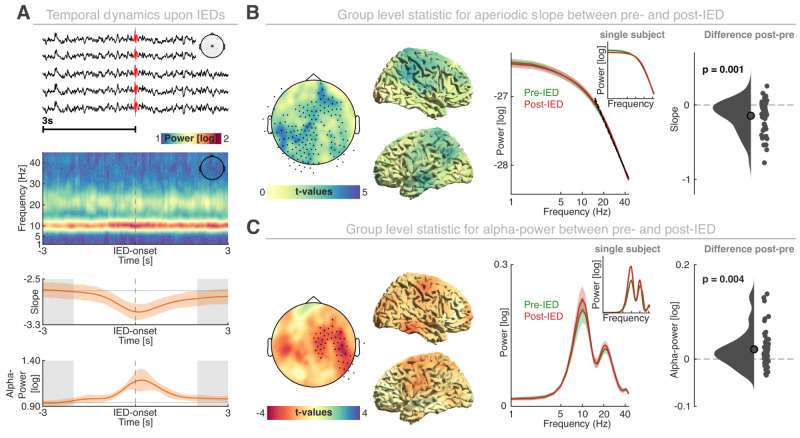
Interictal discharges decrease large-scale hyperexcitability. ***A***, First row, Representative IED (central sensors; inset). Second row, Group-level, perievent, time–frequency representation (central channel; inset). Third row, Time-resolved spectral slope in the same segment. Note the prominent deflection upon the IED (*t* = 0). Fourth row, Time-resolved alpha power. Note the increase after the IED. Colored lines and shaded areas indicate the mean and SEM. The gray-shaded areas delineate 1 s segments before and after the IED that were used for subsequent analyses. ***B***, Group-level statistics for the comparison of spectral slope at sensor- (left; dots represent significant channels) and source-level (center left) from before (−3 to −2 s) and after (2–3 s) an IED. Average aperiodic components across all channels within the significant cluster (center right; the inset depicts a single subject example), along the spectral slope difference between groups (right; dots depict individual patients). ***C***, Group-level statistics for the oscillatory residuals on the sensor- (left) and source-level (center left). The same conventions as in panel ***B***. Mean oscillatory components across all channels within the significant cluster (center right; the inset depicts a single subject example) and the perievent difference in alpha power (right). Note that the *x*- and *y*-axes of all power spectra are log-transformed.

To address the relationship between epileptic discharges, the spectral slope and oscillatory power, we investigated the neural dynamics around isolated IEDs. First, we detected isolated IEDs to compare pre- to postevent dynamics (Fig. 4*A*; *N *= 28.0 ± 1.1; mean ± SEM). Then we statistically compared the spectral slope before and after the IED. We did not consider the IED peak (±2 s) to avoid biasing the spectral estimates by including the sharp IED waveform and subsequent slow-wave activity. We observed a systematic, widespread steepening of the spectral slope after IEDs (Fig. 4*B*; *p* = 0.001; *d* = 0.68; summed *t*_(50) _= 447.98; cluster-based permutation test based on paired *t* tests; pre-IED, −2.87 ± 1.01; post-IED, −3.01 ± 1.00; mean ± SEM), which did not differ significantly between different patient subgroups (*p* = 0.697; *η*^2 ^= 0.05; nonparametric permutation test) or between different medications (*p* = 0.306; *η*^2 ^= 0.12). This observation indicated a decrease in excitability following an IED. This finding was further substantiated by the observation that alpha power reactively increased after an IED (Fig. 4*C*; *p* = 0.004; *d* = 0.44; summed *t*_(50) _= −132.13; pre-IED, 0.20 ± 0. 15; post-IED, 0.22 ± 0.15; mean ± SEM), potentially reflecting a transient increase in functional inhibition to counteract states of hyperexcitability. Again, no significant difference between different patient subgroups (*p* = 0.388; *η*^2 ^= 0.08; nonparametric permutation test) or ASMs (*p* = 0.724; *η*^2 ^= 0.06) was observed.

This spectral slope modulation and the alpha power modulation were not significantly correlated (rho = −0.04; *p* = 0.7843), further supporting the notion that both markers provide unique information. In sum, these observations indicate that IEDs are followed by a transient episode of decreased excitability as indexed by a steepening of the spectral slope and an increase in alpha power. Thus, these results reconcile the seemingly contradictory observations for the direct comparison of IED+ and IED− data (Fig. 3) and demonstrate that IEDs impact systems-level neural excitability.

## Discussion

The present results demonstrate that electrophysiological signatures in whole-head MEG recordings might reflect neural hyperexcitability in patients suffering from GE. IEDs emerge during hyperexcitable brain states and transiently reduce systems-level excitability, which could be interpreted as a mechanism that prevents prolonged states of excessive excitability. Hence, IEDs are not just a reflection of hyperexcitability; rather they might play a crucial role in maintaining the balance between excitation and inhibition, possibly by means of a transient upregulation of inhibitory activity.

In the subset of the patients, genetic testing revealed a specific cellular pathology (e.g., STX1B or SCN1A) as the underlying cause of their epilepsy. The present results further reveal that the spectral slope, a systems-level surrogate marker of neural excitability (Gao et al., 2017; Ahmad et al., 2022; Lendner et al., 2023), indeed captures clinically manifest hyperexcitability.

### The neural basis and functional relevance of aperiodic brain activity

Traditionally, the quantification of electrophysiological activity has focused on rhythmic brain activity, especially alpha oscillations, which constitute the most salient feature in the human M/EEG. Convergent evidence across several lines of inquiry indicated that alpha activity signals functional inhibition (Klimesch et al., 2007; Jensen and Mazaheri, 2010; Palva and Palva, 2011). In contrast to rhythmic brain activity, much less is known about the aperiodic activity, which is often regarded as noise. Based on computational modeling of the local field potential (Gao et al., 2017; Chini et al., 2021), it has been suggested that aperiodic activity as quantified by the slope in the high-frequency band of the double-logarithmic power spectrum might index the balance of excitation and inhibition (*E*/*I* balance), thus providing a critical missing piece to link cellular properties to large-scale brain activity (Blumenfeld, 2003; Ahmad et al., 2022). Recently, the model predictions received empirical support from several lines of inquiry. Increased inhibition can be observed during sleep (Lendner et al., 2020; Bódizs et al., 2021; Kozhemiako et al., 2022; Schneider et al., 2022) and during general anesthesia with propofol or in other states of unconsciousness (Colombo et al., 2019; Lendner et al., 2020). The spectral slope has also been shown to index neural excitability throughout cortical maturation (Chini et al., 2021; Schaworonkow and Voytek, 2021) as well as aging (Voytek et al., 2015; Aggarwal and Ray, 2023) and neurodegenerative processes that are associated with *E*/*I* imbalances (Bush et al., 2023; Wiest et al., 2023). At the cellular level, it had been demonstrated that the spectral slope tracks the overall neural excitability (Lendner et al., 2023), hence indicating that the spectral slope reflects a suitable marker to track hyperexcitability at the whole-brain level. However, to date the precise mechanisms that give rise to aperiodic activity are still actively being debated (Miller et al., 2009; Buzsáki et al., 2012).

A critical shortcoming is that multiple definitions of neural excitability are being used, which depend on the level of observation. Hence, excitability as defined on the cellular level depends on the membrane potential and the likelihood to fire an action potential, which, in turn, is influenced by neurotransmitters, which modulate ion channels (Catterall, 1984; Brodal, 2016). In contrast, neural excitability at the whole-brain level is often indirectly inferred, e.g., from the presence of IEDs (Dahal et al., 2019) or the sensitivity to sensory or magnetic impulses (Pascual-Leone et al., 1998; Paulus et al., 2008). Hence, the interpretation of a surrogate marker as the spectral slope remains difficult (Ahmad et al., 2022).

Here, we studied patients who suffered from a clinically manifest hyperexcitability disorder, which in a subset of patients was the result of a selective mutation in a sodium channel, thus providing the opportunity to study the impact of the mutation on whole-brain dynamics and thereby bridge existing gaps between cellular and systems-level neuroscience. The present results demonstrate that the spectral slope reflects hyperexcitability in GE patients, even when IEDs are momentarily absent. Moreover, the present findings of altered aperiodic activity explain why previous studies reported effects that spanned multiple canonical frequency bands, ranging from the low-frequency delta/theta/alpha bands to the higher-frequency beta/gamma bands (Li Hegner et al., 2018; Stier et al., 2021). Furthermore, the results highlight the merit of spectral decompositions in addition to visual inspection of the time domain data to guide clinical assessment of the M/EEG recordings. Here, we recorded MEG for improved source localization, but the same principles and considerations also apply to EEG recordings (da Silva, 2013). It is critical to highlight that aperiodic activity is potentially sensitive to various artifacts, including muscle artifacts or distortions from sharp transient waveforms (Kozhemiako et al., 2022). Hence, we employed both between- and within-subject comparisons and source localization to mitigate the impact of muscle and artifactual activity. Moreover, we omitted the samples around the IED (Fig. 4), which are characterized by a steepening of the spectral slope. This steepening could also reflect the high amplitude, sharp transient, and not necessarily a genuine shift in the population of *E*/*I* balance. However, the sharp transient would not explain the associated alpha power modulation. Therefore, future studies could employ simultaneous single unit and local field potential recording in order to clarify the impact of firing and waveform sharpness or surrogate excitability markers, either in animal models or in patients suffering from focal epilepsies in the context of invasive EEG monitoring during presurgical evaluation.

### The functional impact of IEDs on electrophysiological correlates of cognition

Hyperexcitability is the defining clinical feature of epilepsy (Stöber et al., 2023), where IEDs hallmark the interictal EEG and are equally prominent in MEG recordings (Kural et al., 2020). At the cellular level, IEDs are associated with burst firing (Hofer et al., 2022). The underlying pathology in generalized epilepsies is often a mutation in voltage-gated sodium channels that gives rise to the hyperexcitability and subsequent IEDs (Catterall et al., 2010; Rusina et al., 2023). Traditionally, IEDs have often been regarded as a mere consequence of the hyperexcitability disorder but might also play a potential protective role and mitigate seizure activity (Chang et al., 2018). Moreover, in recent years, it became evident that IEDs exert an impact on cognitively relevant electrophysiological signatures in a spatially, temporally, and brain-state–dependent manner (Gelinas et al., 2016; Dahal et al., 2019). For example, it had been shown that IEDs engage and potentially hijack hippocampal–neocortical loops that are relevant for memory consolidation, especially during sleep (Beenhakker and Huguenard, 2009; Frauscher and Gotman, 2019). Hence, IEDs often interfere with memory formation and cognitive functioning (Silva et al., 2023). Moreover, IED occurrence is modulated by cognitive effort and engagement. Previously, it had been noted that spectral signatures that typically index cognitive operations, such as alpha and beta activity, might counteract epileptic activity (Vaudano et al., 2017). Here, we replicate and extend these findings by demonstrating that alpha activity might provide the necessary means for functional inhibition in response to IEDs.

Despite the fact that spectral signatures associated with IEDs are well characterized, it remains challenging to predict IED occurrence in M/EEG recordings (Mormann et al., 2007). This difficulty is the direct consequence of the fact that time domain signals are difficult to interpret. The present results now indicate that model-based inference based on the spectral decomposition of the MEG signals might prove beneficial to detect hyperexcitable brain states prior to the IED. A testable hypothesis for future studies is that seizures might be predicted by a flattening of the spectral slope, which is insufficiently counterbalanced by IEDs. It will be of great clinical interest to identify the control mechanisms that predict whether an IED can successfully counterbalance increased excitability (as displayed in Fig. 4) or when it will evolve into a clinically manifest seizure.

It is critical to highlight that we observed IEDs in all patients, who received a variety of ASMs, including valproate, levetiracetam, or lamotrigine (Extended Data Table 1-1). While ASMs are known to suppress IEDs, to date it remains unclear how ASMs influence aperiodic M/EEG activity. From electrophysiological studies that investigated the effects of general anesthesia, it is well-established that administration of propofol (a GABA agonist that is also used for treatment of status epilepticus) steepens the spectral slope, thus indexing the presumed shift toward inhibition (Colombo et al., 2019; Lendner et al., 2020), while, e.g., activating drugs as the NMDA antagonist Memantine have been shown to flatten the spectral slope (Molina et al., 2020). Therefore, it is conceivable that the reported effects are attenuated by ASMs. Thus, larger effect sizes could potentially be observed in the nonmedicated state. Lastly, these present results pave the way for identifying endogenous brain states that naturally suppress epileptic activity. It had long been recognized that IEDs are strongly attenuated during rapid eye movement (REM) sleep (Ng and Pavlova, 2013; Ho et al., 2023). In contrast, sleep deprivation is an effective seizure trigger (Malow, 2004). In line with these clinical considerations, it had recently been reported that the spectral slope in the high-frequency band steepens during REM sleep, while it flattens after sleep deprivation (Lendner et al., 2023), which further suggests that the spectral slope is a useful marker to index neural excitability at the whole-brain level. In sum, these results point toward a dynamic interplay between multiple endogenous mechanisms that counterbalance hyperexcitability. Future studies have to determine whether similar observations can be made in the context of focal epilepsy. A testable hypothesis is that the spectral slope should be flattened near the seizure onset zone, while it might be steeper in remote areas to counterbalance the surplus of excitation (Curot et al., 2023; Johnson et al., 2023).

### Limitations

It is worth noting that our study had some limitations. First, we recruited a heterogeneous patient population that suffered from either IGE, GGE, or GEFS+. We tried to address this heterogeneity by further subdividing the relatively large patient cohort into distinct subgroups. This procedure, however, resulted in a rather small sample size per subgroup which makes it difficult to draw definite conclusions. Future studies need to disentangle the relationship between specific cellular pathologies and aperiodic activity as a potential marker of hyperexcitability in a larger patient cohort.

Moreover, given the cross-sectional study design employed here, we cannot draw causal inferences between the precise cellular pathology and aperiodic activity as a marker for hyperexcitability but only provide correlative evidence for an association between them. Finally, our present study specifically focused on interictal events as periods of hyperexcitability, thus leaving the question unanswered whether aperiodic activity might also track hyperexcitability during ictal episodes.

### Conclusions

Collectively, the present results demonstrate that hyperexcitability in idiopathic or genetic GE, which has its neural basis in pathologies at the cellular level, impacts MEG aperiodic activity. Hence, these results bridge the gap between cellular and systems-level definitions of hyperexcitability and validate recently introduced computational models in a well-characterized clinical cohort. The results might be of potential clinical relevance. For example, quantification of aperiodic activity might provide a noninvasive readout of hyperexcitability even when IEDs are absent (Staba and Worrell, 2014). In the future, aperiodic activity might constitute a traceable biomarker to inform close-looped–responsive neurostimulation to selectively disrupt hyperexcitable brain states and increase inhibition in epileptic circuits (Kundu et al., 2023).

## Synthesis

Reviewing Editor: Arvind Kumar, KTH Royal Institute of Technology Decisions are customarily a result of the Reviewing Editor and the peer reviewers coming together and discussing their recommendations until a consensus is reached. When revisions are invited, a fact-based synthesis statement explaining their decision and outlining what is needed to prepare a revision will be listed below. The following reviewer(s) agreed to reveal their identity: Yevgenia Rosenblum. Note: If this manuscript was transferred from JNeurosci and a decision was made to accept the manuscript without peer review, a brief statement to this effect will instead be what is listed below.

Synthesis

The two reviewers are happy with the revision as most of their concerns have been adequately addressed. However, both have identified several minor edits in the manuscript. So I would like to invite you to submit another version where you have addressed all the remaining minor points.

Reviewer #1

The authors should be commended for their efforts revising their manuscript in line with reviewer feedback. I do have more comments, although they are minor.

- Introduction, lines 91-92: "... in the human magneto- or electroencephalogram (MEG)." The authors mentioned in their rebuttal that they changed all instances of M/EEG to MEG. This is one instance where "M/EEG" is appropriate. It might be worth carefully reviewing the revised manuscript to make sure there are no other similar instances where "MEG" is used inappropriately (e.g., if citing a paper that measured EEG, not MEG).

- Methods, lines 281-283: "All power spectra were initially parameterized using the 'knee' mode, which fits a Lorentzian function with a fixed constant k that indicates the deflection point." This should be "... with a parameter k that indicates ...". The use of "fixed constant" suggests that k is invariant, which is not true for aperiodic mode 'knee'.

Reviewer #2

The authors have addressed all my comments in a satisfactory way. Congratulations on this impressive work. I have only several minor comments:

Abstract:

L52 "At the cellular level, dysfunctional voltage53 gated ion channels constitute a central pathology of GEs" is unnecessary in the Abstract, better remove it and instead (given you will have space) provide some p-values and effect sizes of the results.

L70: "altered MEG background activity" has not been replaced with "aperiodic activity".

L65 "we recorded resting state MEG from male and female GE patients and age-matched healthy controls": please, report the sample size. You could consider replacing it with "we recorded resting state MEG in GE patients (n=__, F=_, age= __) and age-matched healthy controls (n=_).

L 66 "We parametrized the 67 power spectra to": consider replacing it with "We used fooof to", which will give the same amount of words but more important information about the study.

L73: I'd ask to replace "reliably" with "might".

L79 "Based on computational 80 modeling": it is not clear what this means, could you replace it by mentioning the specific method used, please?

L81 "instantaneous": not very clear what you mean by this word, it's not used in your Manuscript, also you explore temporal dynamics afterwards which could not be called "instantaneous".

L82: "hence, providing a novel tool to non-invasively quantify neural excitability at

83 the level of whole-brain recordings": In my opinion, more subtle wording should be used here. For example, "hence, providing a novel non-invasive biomarker that possibly reflects neural excitability at 83 the level of whole-brain recordings".

M/EEG vs MEG terminology: Thank you for replacing this, however, I have to apologize. I see now that I had not elaborated on it enough while sending you my previous Review. My comment wasn't clear enough. It's totally fine to treat EEG and MEG literature together using the "M/EEG" terminology (as in the previous version of your Manuscript) in the Introduction and Discussion. I asked you to replace it with the "MEG" when talking about your specific results (thus, in Methods and Results and Conclusions only) because in your study, you used the MEG method only. I meant that given that you did not measure EEG in your study, it was confusing to use the term "M/EEG" while talking about your specific findings.

L86: the citation could be removed as it appears at the end of the same sentence again.

L93: Could you provide examples of "the variable expression of IEDs", please?

L101: better start a new paragraph. Also, this section (L101 - 116) defines the aperiodic activity, which, however, was already mentioned from lines 95 to 101, i.e., before its description and definition was provided. To facilitate the reading flow, you might consider moving it a bit up.

L 290: "Subsequently, we subtracted the aperiodic spectrum from the raw power spectrum to 291 obtain the oscillatory residuals.": Strictly, speaking the procedure is not as simple as it sounds from this sentence, the procedure involves multiple reiterations and is described in Donoghue et al. with as many as 900 words. So as is, this description is a bit misleading. I'd recommend either removing it (as it is embedded within the FOOOF, so anyway not something that you performed manually) or describing it in a more precise way.

Page 14, Line 2 "Across the patient population": In my opinion, the word "population" is inappropriate here: the results depict the findings in a random sample (of course, we assume that a random sample reflects the general population but it's not equal to it).

Figures: thanks for adding the model fit into Figure 3B, now it's very clear. However, for consistency, I'd recommend adding it to other figures as well.

Knees:

Thanks for adding the information about the knees. However, from theory, we know that when knees are absent, a line could be fitted in log-log space. For none of the Figures you present this does not look like that, i.e., one and only one line could not be fitted, visually it looks like the bend does exist. I'd like to ask you to check your script and its knee parameter definition again and elaborate on this more.

L572 "whole-head MEG recordings capture neural hyperexcitability": please, replace with "whole-head MEG recordings might reflect neural hyperexcitability".

L578: please, add a new paragraph.

L580 "These results": you did not mention any specific results before (in Discussion).

L 599 "double-logarithmic power spectrum might index the balance of excitation and 600 inhibition (E/I-balance": here, "in the high-frequency band" should be added as in Introduction.

L627: please, add a new paragraph.

L704 "In line with these clinical 705 considerations, it had recently been reported that the spectral slope steepens during 706 REM sleep, while it flattens after sleep deprivation (Lendner et al., 2023)": "in the high-frequency band" should be added. This is critical as for the low-frequency band, REM sleep slopes are flatter than SWS slopes.

L736: I'd suggest removing "directly" as you cannot measure if it is indeed directly.

L740: please, replace "provide" with "might provide".
