# Supplementary material for: Aperiodic Activity Indexes Neural Hyperexcitability in Generalized Epilepsy
Source: eNeuro. 2024 Sep 3;11(9):ENEURO.0242-24.2024. doi: 10.1523/ENEURO.0242-24.2024 (PMC11376430; doi:10.1523/ENEURO.0242-24.2024)
Supplement: Table1-1 — Patient characteristics Overview of the included patient cohort, which specifies sex, age, diagnosis, medication status and time from the last seizure. Abbreviations: GGE: genetic generalized epilepsy; IGE: idiopathic generalized epilepsy; GTCS: generalized tonic clonic seizures; JAE: juvenile absence epilepsy; JME: juvenile myoclonic epilepsy; FC: febrile convulsions; SE: status epilepticus; LEV: Levetiracetame; LTG: Lamotrigine; VPA: Valproate; ESX: Ethosuximide; ESL: Eslicarbazepine; TPM: Topiramate; STP: Stiripentol; CLB: Clobazam. Download Table1-1, DOCX file. [file eneuro-11-ENEURO.0242-24.2024-s001.docx]

**Extended Data Table 1-1**

| **Patient** | **Sex** | **Age** | **Diagnosis** | **Medication** | **Last Seizure** |
| --- | --- | --- | --- | --- | --- |
|  |  |  |  |  |  |
| 1 | f | 43 | GGE with GTCS and Absences | ESL 2000mg, LEV 2000mg/d | 21 months |
| 2 | f | 24 | JME with GTCS and Myoclonic Seizures | LEV 1000mg/d | > 3 years |
| 3 | f | 18 | STX1B with FC, GTCS | N/A | N/A |
| 4 | f | 29 | IGE with Absences and GTCS | None | > 10 years |
| 5 | f | 20 | IGE with Absences | LEV 1000mg/d | Daily |
| 6 | f | 35 | GGE with Myoclonic Seizures, Absences and GTCS | VPA 1000mg/d, LEV 3000mg/d | 29 months |
| 7 | f | 18 | JME with Myoclonic Seizures and GTCS | LTG 200mg/d | 3-6 months |
| 8 | f | 24 | IGE with GTCS | VPA (unknown dosage) | N/A |
| 9 | f | 23 | IGE with GTCS | LTG 250mg/d | > 5 years |
| 10 | f | 16 | STX1B with FC, GTCS | N/A | N/A |
| 11 | f | 22 | IGE with GTCS | None | Weekly |
| 12 | m | 43 | STX1B with Absences and GTCS | N/A | < 5 years |
| 13 | f | 14 | STX1B with GTCS | LTG 400mg/d, VPA 300mg/d | < 6 months |
| 14 | f | 32 | JAE with Absences and GTCS | LTG (unclear dosage) | N/A |
| 15 | m | 27 | IGE with GTCS | VPA 1200mg/d, LEV 1000mg/d | 2 months |
| 16 | m | 30 | JAE with Absences and GTCS | VPA 2000mg/d, LEV 1000mg/d | N/A |
| 17 | f | 36 | JAE with Absences and GTCS | VPA 1000mg/d | > 3 years |
| 18 | f | 18 | JAE with Absences and GTCS | LTG 300mg/d, LEV 1500mg/d, ESX 750mg/d | Weekly |
| 19 | f | 64 | STX1B with FC, Absences | None | N/A |
| 20 | m | 21 | JME with FC, GTCS | VPA 1000mg/d | N/A |
| 21 | f | 18 | IGE with Absences and GTCS | VPA 750mg/d | 6 months |
| 22 | m | 31 | SCN1A with GTCS | unknown | N/A |
| 23 | m | 16 | STX1B with Absences, Myoclonic Seizures, GTCS | VPA 1200mg/d, LEV 2000mg/d | Weekly |
| 24 | f | 18 | STX1B with FC | N/A | N/A |
| 25 | m | 21 | STX1B with FC, GTCS | N/A | N/A |
| 26 | f | 49 | JAE with Absences and GTCS, refractory SE | VPA 900mg/d | 1 year |
| 27 | f | 48 | STX1B with FC | N/A | N/A |
| 28 | m | 44 | STX1B with Absences and FC | N/A | N/A |
| 29 | m | 20 | SCN1A with Absences, Myoclonic Seizures, GTCS and conv. SE | STP 2000mg/d, TPM 200mg/d, VPA 600mg/d, CLB 10mg/d | Monthly |
| 30 | m | 7 | STX1B with FC | N/A | N/A |
| 31 | m | 20 | STX1B with FC | N/A | N/A |
| 32 | m | 18 | JAE with Absences | None | Monthly |
| 33 | m | 43 | SCN1A with Absences, GTCS and FC | N/A | N/A |
| 34 | f | 25 | JME with Myoclonic Seizures, GTCS | LEV 2500 mg/d | 2 years |
| 35 | m | 42 | STX1B with FC | None | N/A |
| 36 | m | 37 | Jeavons Syndrome, Eyelid Myoclonia, GTCS | LEV 2000mg/d | 2 years |
| 37 | m | 22 | JME with Myoclonic Seizures, GTCS | LEV 1000mg/d | N/A |
| 38 | f | 24 | GGE with Absences, Eyelid Myoclonia, Myoclonic Seizures, and GTCS | VPA 900mg/d, ESX 750mg/d | 3 years |
| 39 | m | 21 | IGE with GTCS | LEV 3000mg/d | < 6 months |
| 40 | m | 42 | JME with Myoclonic Seizures and GTCS | LEV 3000mg/d, VPA 600mg/d | N/A |
| 41 | m | 43 | STX1B with Absences | N/A | N/A |
| 42 | f | 49 | GGE with Absences and GTCS | LTG 300mg/d | N/A |
| 43 | f | 18 | GGE with GTCS | LTG 500mg/d | > 2 years |
| 44 | f | 25 | GGE (unclassified) | LEV 1000mg/d | N/A |
| 45 | f | 56 | STX1B with FC | N/A | N/A |
| 46 | m | 36 | JAE with Absences and GTCS | LEV 1000mg/d | Monthly |
| 47 | f | 25 | JAE with Absences and GTCS | LTG 600mg/d, ESX 500mg/d | N/A |
| 48 | f | 32 | JAE with Absences and GTCS | LEV 1000mg/d | N/A |
| 49 | f | 24 | JAE with Absences and GTCS | None | > 10 years |
| 50 | m | 19 | Dravet Syndrome (p.I415K), FC, GTCS | TPM 225mg/d, VPA 2400mg/d | Monthly |
| 51 | m | 28 | IGE with Absences, Myoclonic Seizures, GTCS | LEV 1000mg/d | > 1 year |

**Patient characteristics.**

Overview of the included patient cohort, which specifies sex, age, diagnosis, medication status and time from the last seizure. Abbreviations: *GGE*: genetic generalized epilepsy; *IGE*: idiopathic generalized epilepsy; *GTCS*: generalized tonic clonic seizures; *JAE*: juvenile absence epilepsy; *JME*: juvenile myoclonic epilepsy; *FC*: febrile convulsions; *SE*: status epilepticus; *LEV*: Levetiracetame; *LTG*: Lamotrigine; *VPA*: Valproate; *ESX*: Ethosuximide; *ESL*: Eslicarbazepine; *TPM*: Topiramate; *STP*: Stiripentol; *CLB*: Clobazam
